# Microfluidic Wound-Healing Assay for ECM and Microenvironment Properties on Microglia BV2 Cells Migration

**DOI:** 10.3390/bios13020290

**Published:** 2023-02-17

**Authors:** Ehsan Yazdanpanah Moghadam, Nahum Sonenberg, Muthukumaran Packirisamy

**Affiliations:** 1 Optical-Bio Microsystems Laboratory, Micro-Nano-Bio Integration Center, Department of Mechanical and Industrial Engineering, Concordia University, Montreal, QC H3G 1M8, Canada; 2 Department of Biochemistry, Goodman Cancer Research Center, McGill University, Montreal, QC H3A 1A3, Canada

**Keywords:** microfluidic wound-healing migration assay, cell migration, microglia cells, extracellular matrix coating, substrate rigidity

## Abstract

Microglia cells, as the resident immune cells of the central nervous system (CNS), are highly motile and migratory in development and pathophysiological conditions. During their migration, microglia cells interact with their surroundings based on the various physical and chemical properties in the brain. Herein, a microfluidic wound-healing chip is developed to investigate microglial BV2 cell migration on the substrates coated with extracellular matrixes (ECMs) and substrates usually used for bio-applications on cell migration. In order to generate the cell-free space (wound), gravity was utilized as a driving force to flow the trypsin with the device. It was shown that, despite the scratch assay, the cell-free area was created without removing the extracellular matrix coating (fibronectin) using the microfluidic assay. It was found that the substrates coated with Poly-L-Lysine (PLL) and gelatin stimulated microglial BV2 migration, while collagen and fibronectin coatings had an inhibitory effect compared to the control conditions (uncoated glass substrate). In addition, the results showed that the polystyrene substrate induced higher cell migration than the PDMS and glass substrates. The microfluidic migration assay provides an in vitro microenvironment closer to in vivo conditions for further understanding the microglia migration mechanism in the brain, where the environment properties change under homeostatic and pathological conditions.

## 1. Introduction

Cell migration, as a fundamental process, plays an essential role in tissue engineering, wound healing and the immune response [1,2]. During cell migration, cell mobility is correlated with the properties of the substrate, including chemical properties, such as the extracellular matrix (ECM) [3,4], and physical substrate properties, such as rigidity and topography [5,6]. Microglial cells are the innate immune cells in the central nervous system (CNS). They are highly active and migratory in brain development (such as neurite formation) and pathological conditions (such as the pathogenesis of many neurological diseases) [7,8,9]. Microglia cells in migration encounter ECMs and various substrate elasticities (stiffness) of CNS tissue, which change in aging [10,11,12], brain pathologies [12,13,14], neuroinflammation [13,14,15], cerebral neoplasia [15] and neurodegenerative diseases [16,17]. The substrate characteristic is an essential part of brain development and disease progression, which is modeled with ECM coatings [4] and biocompatible substrates such as polydimethylsiloxane (PDMS) in vitro [17,18,19,20]. Therefore, a study on the substrate effect on microglia cell migration helps the understanding of cell mobility in pathophysiological conditions.

It was shown that the microglia cell migration changed in response to the variation of their physical environment properties. The cells were significantly activated to migrate towards the stiffer neural implant in the rat brain and an increase in the inflammatory microglial marker. However, under exposure to the soft neural implant, the microglia cells showed more compatibility by decreasing their reactions against the foreign body [21]. The microglia cells preferentially migrated toward the stiffer region when exposed to the substrate with the stiffness gradient. It showed that physical signals, similar to chemical signals, affect microglial attraction in the brain [22]. It was reported that PDMS substrates with different elasticities were utilized to assess microglial characteristics and functionalities. Substrates with less elasticity mimicking the brain did not change the cell viability, thus enhancing cell proliferation [18]. The majority of research regarding microglia cells has been carried out on the impact of soluble factors, such as chemokines, cytokines, and cell-cell interaction. However, the migratory effect on the chemical and physical properties of the substrate in interaction with microglia cells is not fully understood [18].

To quantify the collective cell migration, the scratch assay (wound-healing assay) is a regular approach [23,24,25]. This method introduces a cell-free area by scratching over a confluent cell monolayer with a pipette tip. The cell migration was characterized by imaging the cell movement to occupy the cell-free region. The scratch assay is easy to use, inexpensive, and only requires standard equipment in the laboratory [26]. Liberio et al. [27] utilized the scratch assay to characterize the migration of prostate cancer cells (LNCaP cells) on ECM coatings, including poly-L-lysine, poly-L-ornithine, collagen type IV, fibronectin and laminin. It was shown that only laminin simulated cell migration, while the migration on other ECM coatings was less than in the control cells. In addition, the scratch assay assessment of Schwann cells on ECM coatings showed that laminin, poly-L-lysine and poly-L-ornithine inhibited cell migration compared to the control cells. In contrast, collagen type I and fibronectin provoked Schwann cell migration [28]. However, in the scratch assay, the cells at the edge are damaged, which negatively affects cell migration [29,30]. In addition, the ECM over the substrate is removed by scratching [31]. The scratch over the soft substrate, such as PDMS, makes a rugged surface for the cells. The wound-healing barrier and microfluidic wound-healing device assays have been proposed to overcome these shortcomings for investigating the cell migration on different substrates. The wound-healing barrier was reported to study the cell migration of Human kidney tubular epithelial (HKC-8) cells on collagen and fibronectin coatings. It was revealed that fibronectin promoted HKC-8 cells more than collagen IV [31]. Another investigation, performed by Lin et al. [32], showed that fibroblast cell migration was stimulated on gelatin more than on poly-L-lysine coatings using the wound-healing barrier. However, the barriers do not allow for the investigation of cell migration under the fluid flow. Microfluidic assays have been developed as an alternative platform to optimize wound-healing migration assays [33,34,35]. They enable the consumption of fewer reagents than other assays and have high controllability in the fluid flow and flexibility in the design, making these microassays capable of providing the cells with conditions more akin to the in vivo microenvironment. For instance, to study human lung fibroblasts cell (HLF) migration on substrates with different physical properties, including glass, polystyrene, PDMS and polymethyl methacrylate (PMMA), Lin et al. [36] used a developed wound-healing microfluidic assay. In this method, laminar trypsin and cell media layers were flowed within the device by a syringe pump. The cells exposed to the trypsin were washed to generate the cell-free gap. It was found that the HLF cells had the highest migration rate and distance on polystyrene, whereas PMMA was the lowest. To facilitate the wounding process through a microfluidic wound-healing migration assay, gravity was applied as the driving force instead of the syringe pump to flow the trypsin over the confluent vascular smooth muscle cells [37]. However, there is no report to assess microglial BV2 cell migration on the substrates coated with ECMs and conventional bio-applicant substrates using wound-healing assays.

Here, a microfluidic wound-healing migration assay was proposed to study the microglia BV2 migration on a wide range of substrates with various chemical and physical properties. This micro-migration assay was capable of generating a cell-free area by creating a laminar trypsin flow over the confluent cell monolayer. The trypsin was flown by gravity force without using the syringe pump and microfluidic tubing. Despite the scratch assay, it was shown that the device enabled wounding over the confluent cell monolayer without removing the extracellular matrix. Microglia BV2 migration was studied on the substrate coated with collagen, fibronectin, PLL and gelatin, as well as the substrate with various physical properties, including polystyrene, PDMS and glass. The current investigation helps to create a microenvironment closer to in vivo conditions to obtain reliable results on cell migration.

## 2. Materials and Methods

### 2.1. Device Design and Fabrication

**Figure 1:**
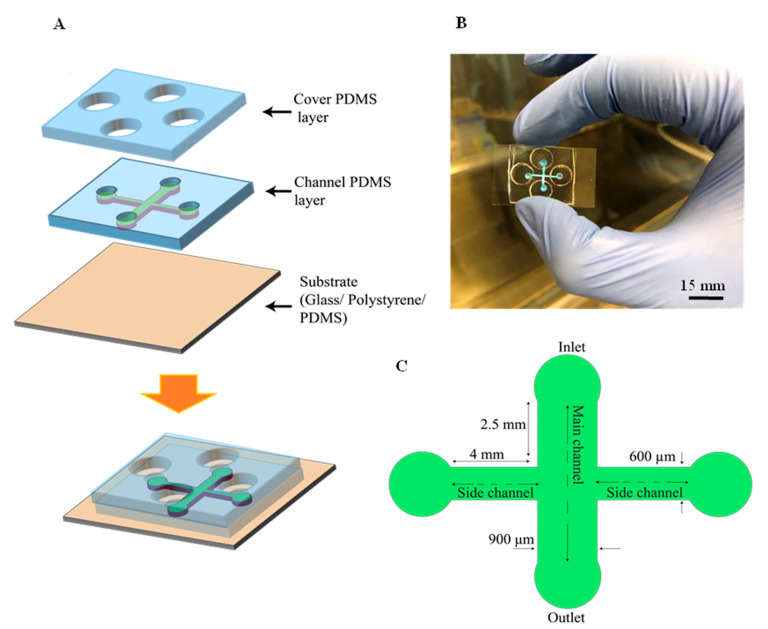
Schematic of the microfluidic device. (**A**) exploded view of the two PDMS layers that bonded on the substrate. (**B**) Actual photography of the microfluidic device with the microchannels filled with red dye, 3D view, scale bar = 15 mm. (**C**) Geometrical configuration, top view.

**Figure 2:**
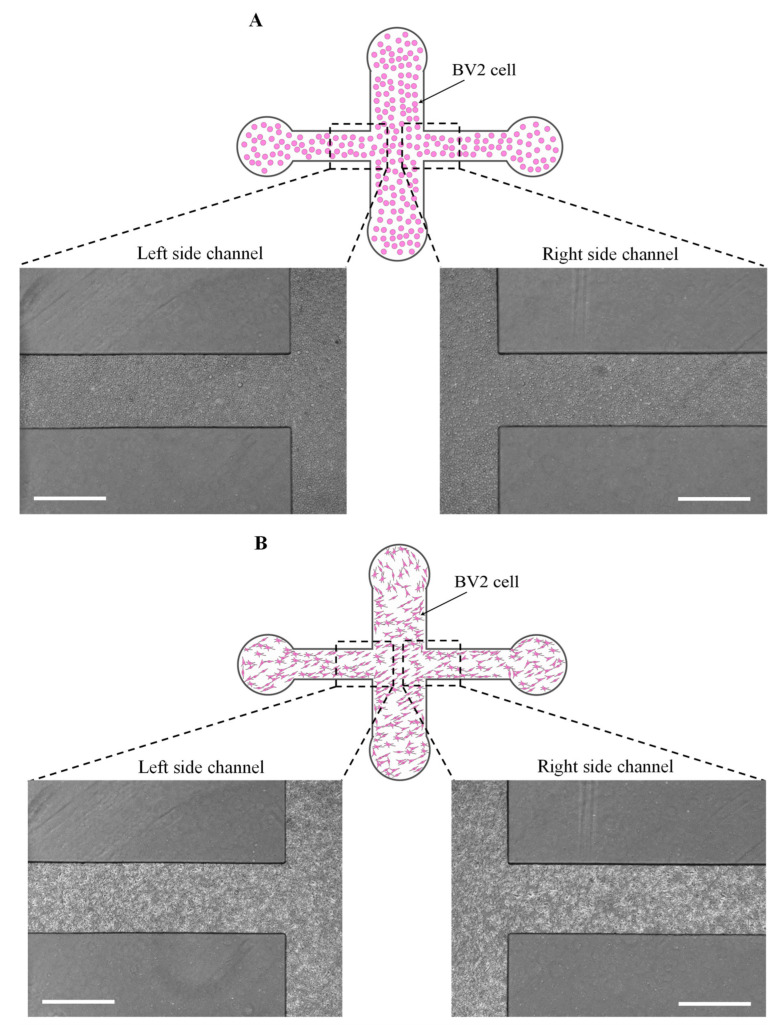

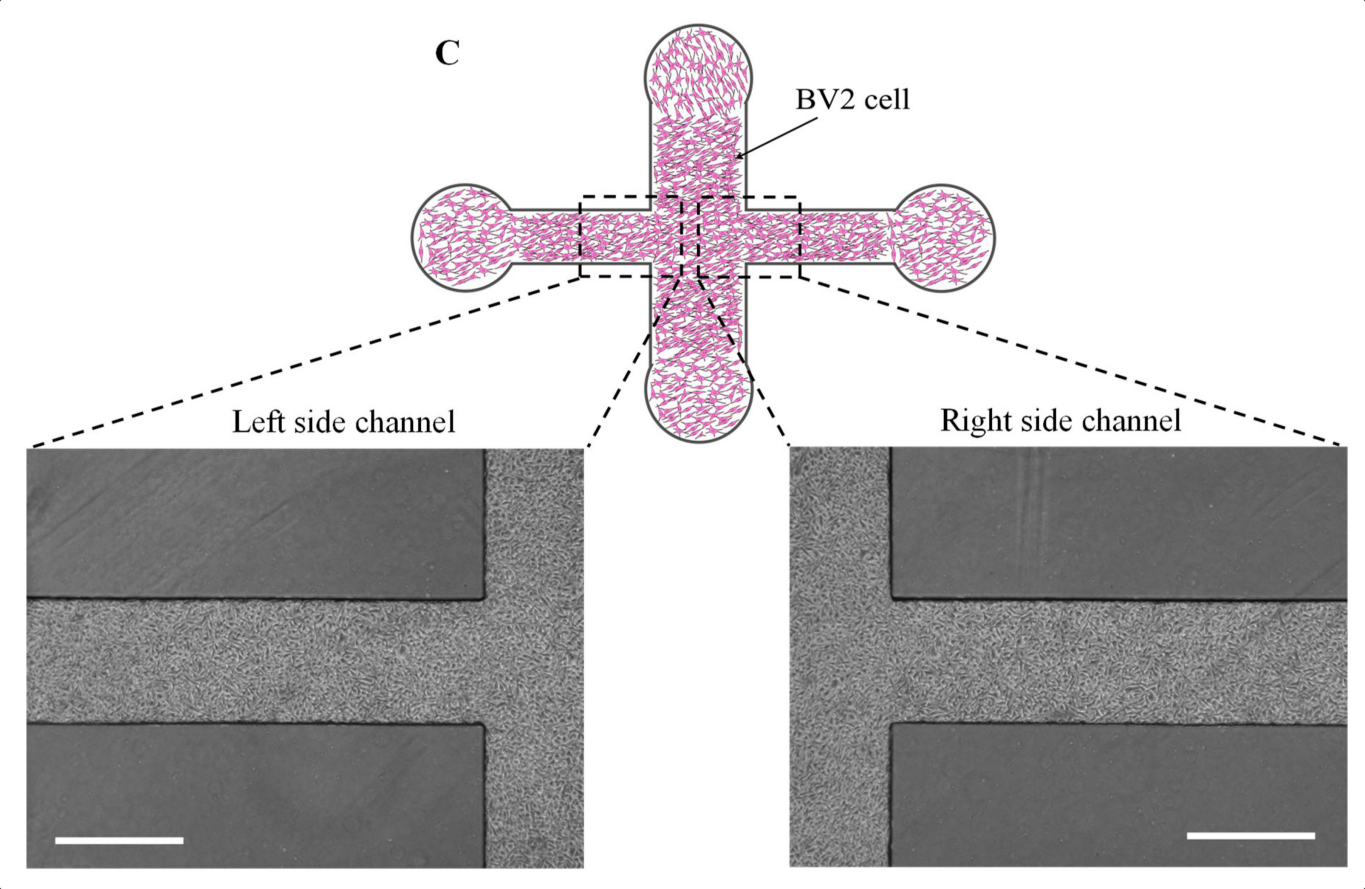
Cell seeding to cell confluence in the microfluidic wound-healing assay. (**A**) cell loading, (**B**) cells attached to the substrate after 40 min, (**C**) confluency of the cells after 12 h, scale bar = 600 µm.

The device has two PDMS layers (channel and cover layer) on the substrates of polystyrene, glass and PDMS (Figure 1A,B). The channel PDMS layer (4 mm in height) includes the main channel (width = 0.9 mm, length = 5.6 mm and height t = 100 µm) and side channels (width = 600 µm, length = 4 mm and height = 100 µm) (Figure 2C). The cover PDMS layer (2.5 mm in height) contains four reservoirs (8 mm in diameter). The microfluidic device was fabricated using the standard soft-lithography technique with a replica molding of PDMS. To make a master mold on the silicon wafer, a negative photoresist (SU8-2075, MicroChem, Round Rock, TX, USA) was patterned by photolithography. A prepolymer of PDMS mixed with the elastomer curing agent (10:1 ratio, Sylgard 184, Dow Corning, Midland, MI, USA) was poured over the molds. After curing the PDMS for 2 h at 70 °C, the PDMS was peeled off from the master mold. In the channel PDMS layer, the terminals of each channel were punched to make circular ports (3 mm in diameter) (Figure 1A,C). Four media reservoirs in the cover PDMS layer were punched with a biopsy punch (Figure 1A). The top and bottom sides of the channel PDMS layer were bonded to the cover PDMS layer and the glass coverslip or PDMS substrates, respectively, by treating with oxygen plasma for 90 s. However, the polystyrene substrate (Falcon 353003 Cell Culture Dish, Corning Inc., New York, NY, USA) was bonded to the channel PDMS layer according to the previously reported procedure [38]. Briefly, the petri dish was treated with oxygen plasma, and a 5% (3-Aminopropyl) triethoxysilane (APTES, Sigma, Burlington, MA, USA) solution was poured to cover the petri dish surface for 5 min. The APTES solution was washed with DI water. After drying the petri dish, the PDMS treated with oxygen plasma was bonded to the petri dish substrate. The petri dish was kept in the oven for 30 min at 65 °C.

### 2.2. Surface Coating

The experiment was performed according to the manufacturer’s protocol. In order to coat the device with glass cover slide substrates, a collagen type I solution from rat tail (0.01 mg/mL diluted with PBS, Sigma, Burlington, MA, USA), PLL (0.01 mg/mL, Sigma, Burlington, MA, USA), gelatin (0.01 mg/mL diluted with PBS, Fisher Scientific, Hampton, NH, USA),and human plasma fibronectin (0.01 mg/mL diluted with PBS, Sigma, Burlington, MA, USA) were loaded into the device. The devices were incubated with collagen, PLL and gelatin in the hood or overnight for human plasma fibronectin for 30 min. Then, the devices were washed with PBS to be prepared for cell seeding.

### 2.3. Cell Culture and Cell Seeding in the Microfluidic Device

The murine microglial BV2 cells (~2 × 10^5^ cells/mL concentration) were cultured in Dulbecco’s Modified Eagle’s Medium (DMEM; Wisent Technologies, Canada), supplemented with 10% fetal bovine serum (FBS, Invitrogen) and 1% Penicillin-streptomycin (Wisent Technologies, Canada). The cells were incubated at 37 °C in 5% CO_2_ until they reached 90% confluence. After harvesting the cells with 0.05% trypsin (Wisent Technologies, Saint-Bruno, QC, Canada), the cells were centrifuged at 1100 rpm for 10 min. Then, the supernatant was removed, and new cell media (1 mL) was added to resuspend the cells with a cell concentration of 1 × 10^7^ cells/mL. For cell seeding with a high density in the device, the cells were seeded as follows: 100 µL from the cell stock were pipetted into the device from the inlet port. Then, four 200 µL pipette tips (pipette tips blocked with PDMS) were inserted into the outlet port, side channel ports, and the inlet port to close the ports. This technique helped to stop the flow into the device, and the cells were trapped within the device (Figure 2A). After 40 min of the device incubation at 37 °C in 5% CO_2_, the cells adhered to the substrate (Figure 2B). The pipette tips were removed gently; next, 80 µL of fresh cell media was added to the reservoirs.

### 2.4. Experimental Setup for Wound Creation

**Figure 3:**
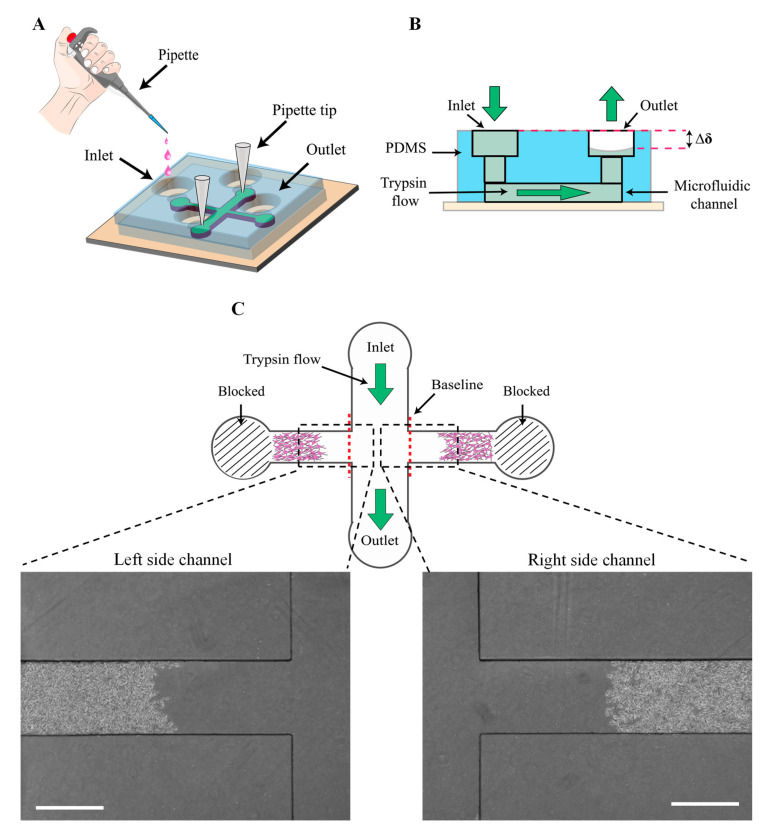
Process of cell-free generation. (**A**) Experiment setup. While the terminals of the side channels were blocked, trypsin was added to the inlet. (**B**) Side view and (**C**) top view, gravity as a driving force was used to flow the trypsin from the inlet to the outlet to generate the cell-free area in the main channel.

The cell-free area was generated after 12 h from the cell seeding when the cells in the device became confluent. Each experiment was performed as follows. Step 1: the media in the reservoirs was aspirated. Step 2: the pipette tips were inserted into the side channels’ ports. Step 3: 200 µL and 50 µL of 0.25% trypsin (Wisent Technologies, Saint-Bruno, QC, Canada) were added to the inlet and outlet reservoirs, respectively. Step 3: After 6 min, when the cells in the main channel were removed, the trypsin was aspirated from the inlet and outlet reservoirs, and then the trypsin was neutralized by adding 80 µL media to the inlet reservoir. Step 4: the pipette tips were removed gently, and 80 µL media was added to the reservoirs. Every 12 h, the media was changed (Figure 3A–C).

### 2.5. Immunoassay for Fibronectin

**Figure 4:**
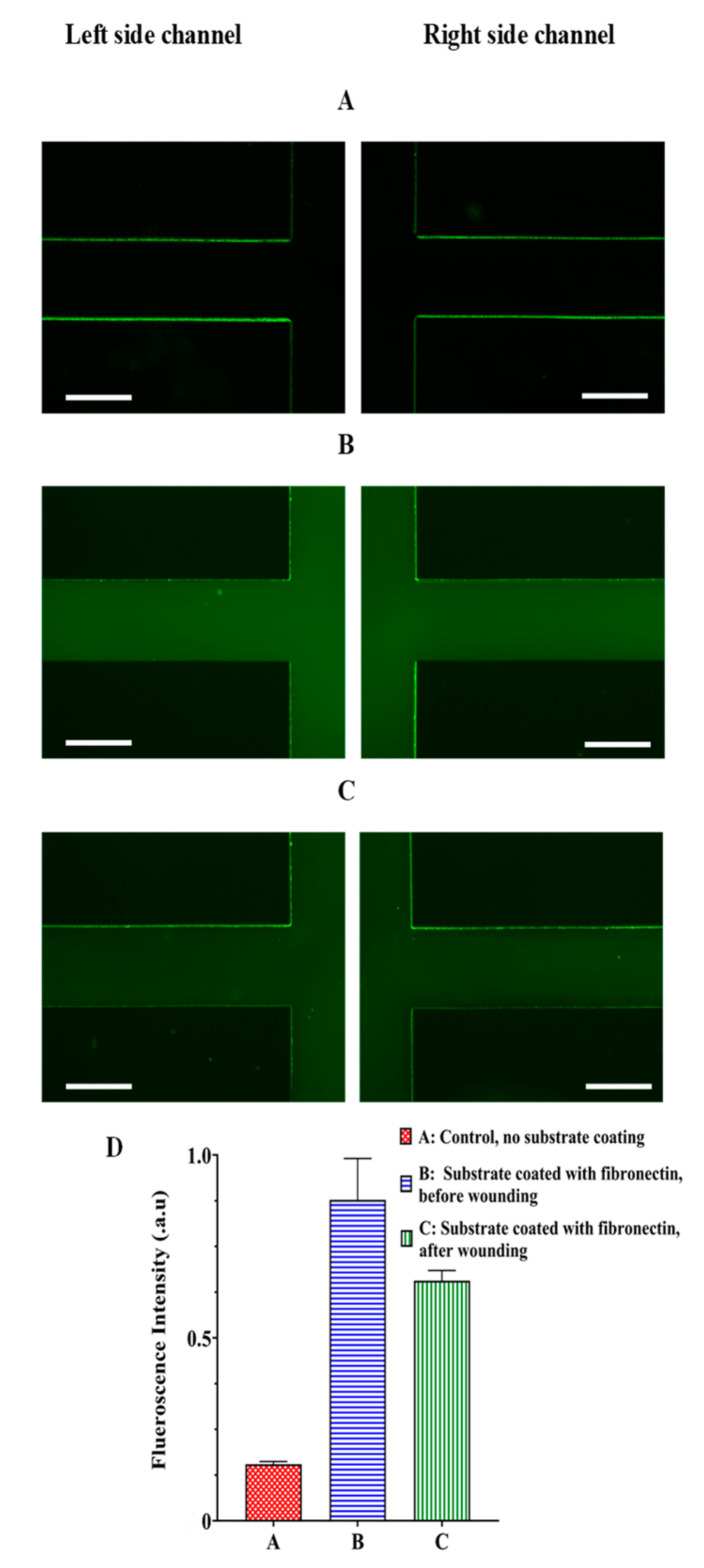
Fluorescence images from the device (**A**) glass substrate without coating (the control condition), (**B**) glass substrate coated with fibronectin before performing the process of the cell-free area creation (no flowing within the device), and (**C**) glass substrate coated with fibronectin after generating the cell-free area. (**D**) Quantification of the fluorescence intensity in the side channels in cases A, B and C, scale bar = 600 µm.

An immunoassay was used to evaluate the surface coated with fibronectin in two side channels once the cell-free area was generated. The device substrate coated with fibronectin was washed with PBS and 5% bovine serum albumin (BSA) was added and incubated at room temperature for 1 h. After rinsing with PBS, the rabbit fibronectin antibody (1:400 diluted with PBS, Novusbio) was loaded within the device and incubated at room temperature for 2 h. After washing with PBS, the secondary antibody, Alexa Fluor^®^ 488, goat anti-rabbit IgG (1:500 diluted with PBS, Invitrogen), was incubated at room temperature for 1 h. Next, the device was rinsed with PBS to take the fluorescence images with a fluorescence microscope (4x objective lens, AMG EVOS FL) [39]. The fluorescent images were captured for three cases: the uncoated substrates (control, Figure 4A) and coated substrates with fibronectin before (Figure 4B) and after wounding (Figure 4C). The image J 6.0 software was used to measure the fluorescent intensity of the images in three cases, including the uncoated surface and the coated surface before and after wounding. The average fluorescent intensity of the main channel was quantified to evaluate the surface coating. The average fluorescent intensity in each case was normalized to the maximum fluorescent intensity in the coated surface before wounding.

### 2.6. Assessment of Cell Migration

The images were acquired to assess the cell migration at 0 h, 12 h, 24 h and 48 h under the fluorescence microscope (AMG EVOS FL) at a magnification of 4x objective lens. The image J 6.0 software was utilized to quantify the cell-free area, defined as the area from the cell wound edge to the baseline (Figure 3C). The cell-free area quantified for each device at any time was the average cell-free area of the left and right side channels. The cell migration area was the substrate of the cell-free area at the desired time (A_t_) from the initial cell-free area (A_0_). To measure the average cell migration distance at each time point, the cell migration area at the desired time was divided into the side channel width. The migration rate (average cell migration speed) was calculated by dividing the average cell migration distance at each time point to the experiment duration [36].

### 2.7. Statistical Analysis

The data are presented as the mean ± standard deviation (SD). For each condition, five independent experiments were performed (*n* = 5). Statistical significances were analyzed by ANOVA using the GraphPad PRISM software. *p* < 0.05 was considered to show a statistically significant difference.

## 3. Results and Discussion

### 3.1. Mechanism of the Wound Generation Using the Microfluidic Chip

We presented a microfluidic wound-healing assay to characterize microglia BV2 cell migration on the substrates under different physical and chemical cues. In order to generate the cell-free area, first, the terminals of the side channels were blocked to increase the pressure in the side channels compared to the main channel, leading to the laminar trypsin flow directed along the main channel. Then, the laminar trypsin was driven in the main channel using gravity when the hydrostatic pressure between the inlet and outlet reservoirs was created by adding 200 µL and 50 µL trypsin in the inlet and outlet, respectively. In addition, before flowing the trypsin, there was cell media inside the microchannels. When the trypsin moved from the inlet to the outlet, the initial cell media in the side channels was trapped. It helps to neutralize the trypsin diffusion to the side channels, leading to fewer cells being removed.

In this approach, due to the use of gravity as a driving force for flowing the trypsin, it is not required to provide technical equipment, such as microfluidic tubing, a syringe pump and pneumatic valves. Therefore, this approach makes cell migration assessment affordable and facilitative in the wound-healing method.

### 3.2. Evaluation of Surfacing Coating

An immunoassay for the surface coated with fibronectin was used to evaluate the surface coating after cell-free area generation. Figure 4 demonstrates the fluorescent images from the uncoated device substrate (Figure 4A) and coated substrate with fibronectin before (Figure 4B) and after (Figure 4C) cell-free creation. As shown in Figure 4D, although the fluorescent intensity for the surface coated after wounding was less than before wounding, there was a significant enhancement of the fluorescent intensity for the surface coated after wounding compared to the uncoated surface. Therefore, the result showed that, despite the scratch assay [31], our method of creating the cell-free area did not remove ECM coating. It enables us to quantify the cell migration in a microenvironment closer to in vivo conditions.

### 3.3. Effect of Physical Properties of Substrates on Microglial BV2 Cells Migration

**Figure 5:**
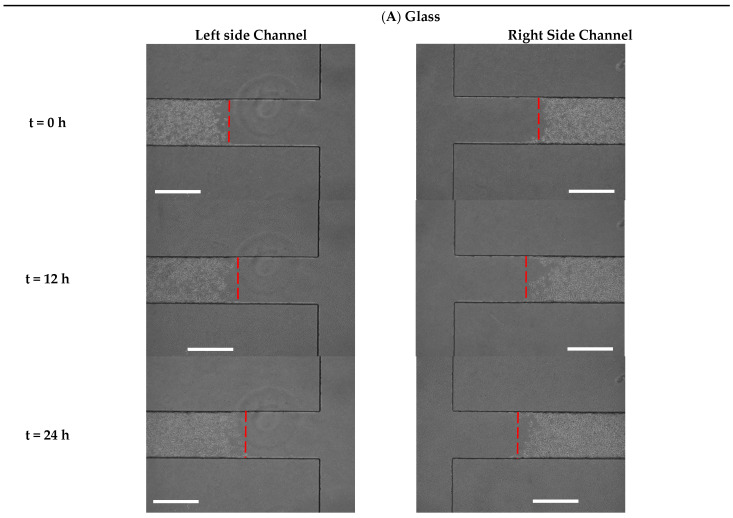

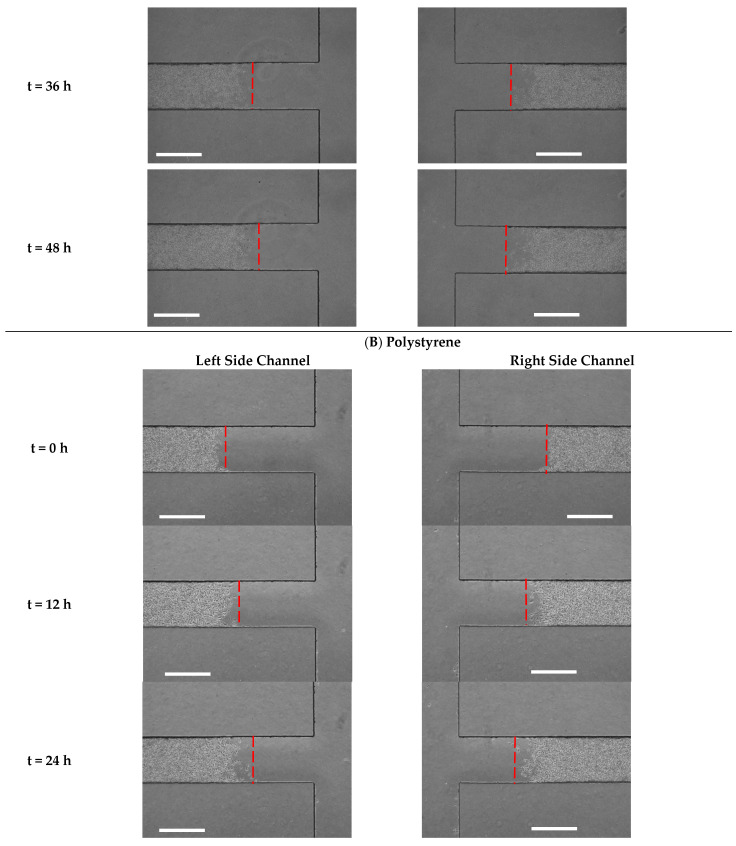

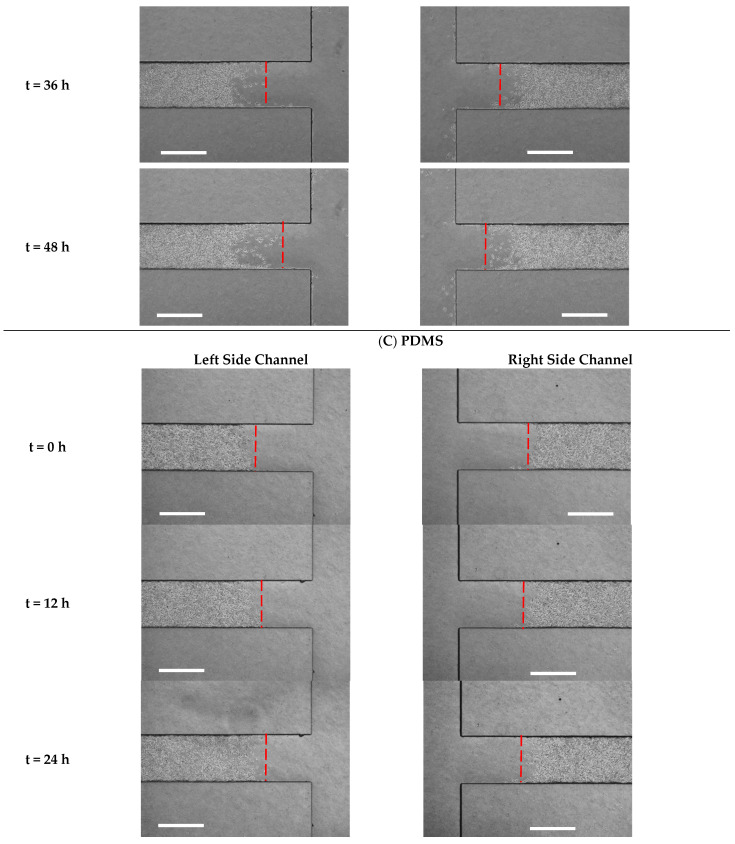

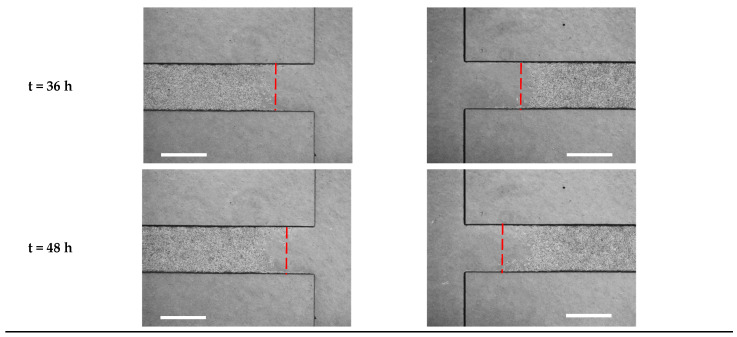
Time-lapse images from cell migration on the substrates of (**A**) glass, (**B**) polystyrene (**C**) PDMS after 0, 12, 24, 36 and 48 h, scale bar = 600 µm.

**Figure 6:**
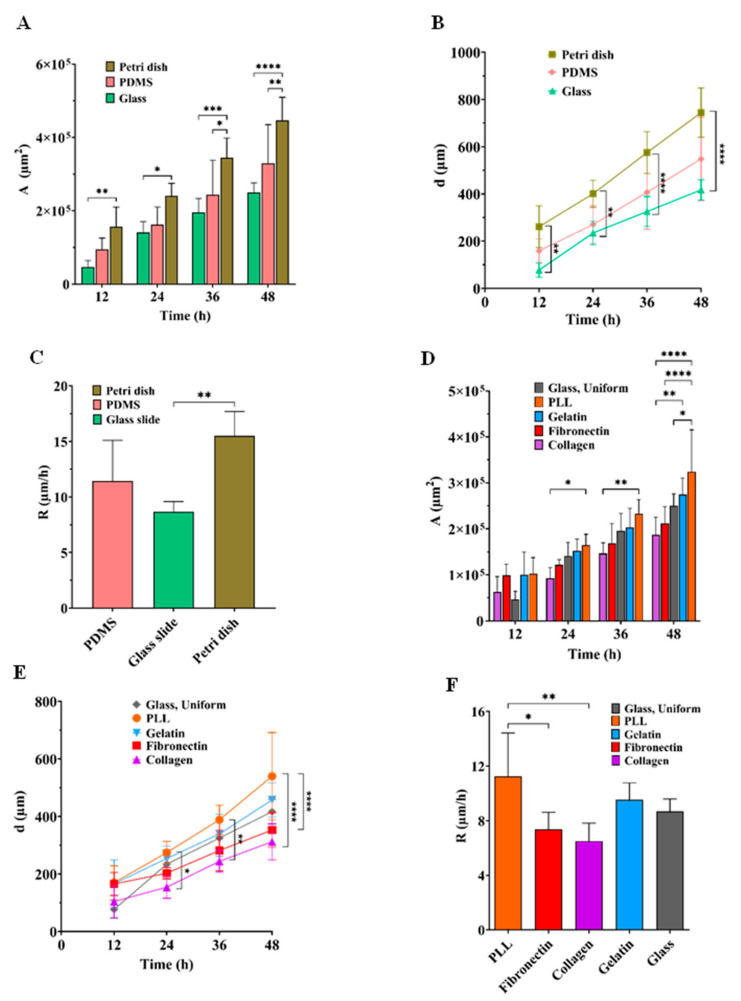
Quantification of cell migration on glass, polystyrene and PDMS substrates. (**A**) cell migration area and (**B**) distance substrates at 0 h, 12 h, 24 h, 36 h and 48 h after creating the cell-free area and (**C**) migration rate of the cells after 48 h. Quantification of cell migration on glass (control), PLL, fibronectin, collagen and gelatin-coated substrates. (**D**) cell migration area and (**E**) distance at 0 h, 12 h, 24 h, 36 h and 48 h after generating the cell-free area, and (**F**) migration rate of the cells after 48 h. Value was shown as mean ± SD. For each condition, five independent experiments were performed (*n* = 5). *: *p* < 0.5, **: *p* < 0.01, ***: *p* < 0.0001 and ****: *p* < 0.0001.

To characterize the effect of the physical properties on microglial BV2 cell migration, we utilized glass, polystyrene and PDMS (Figure 5A–C) as conventional substrates used in bio-applications. As indicated in Figure 6A, after 48 h of wounding, polystyrene had the highest stimulatory effect on BV2 cell migration (446,716.8 ± 6272.5 µm^2^) compared to PDMS (329,056.8 ± 105,873.4 µm^2^) and glass (250,106 ± 26,016 µm^2^). It was found that after 48 h of creating the cell-free area, the BV2 cell migration distance on the polystyrene, PDMS and glass was 744.52 ± 104.73 µm, 548.42 ± 176.45 µm, 416.84 ± 43.36 µm, respectively (Figure 6B). As shown in Figure 6C, over 48 h, the microglial BV2 cells migrated with a migration rate of 15.51 ± 2.18 µm/h on the polystyrene, which was more significant than on the PDMS (11.43 ± 3.67 µm/h) and glass (8.68 ± 0.93 µm/h). Accordingly, among the conventional bio-applicant substrates, microglia BV2 cells moved on the polystyrene at a higher rate than on the PDMS and glass. This is because the polystyrene used in our experiment was treated with oxygen-containing plasma, which oxidized the surface to enhance the cell spread [40,41]. There was supportive evidence when Lin et al. [36] showed that the migration of human lung fibroblast cells on the polystyrene substrate was higher than on glass and PDMS using a wound-healing microfluidic migration assay. In addition, in comparing the cell migration on the PDMS and glass substrates, our findings revealed that microglia BV2 cells moved faster on the substrate with less stiffness (PDMS). Cell migration is a balance between cell attachment and release from the substrate. Cells on the stiffer (glass) substrate were attached more strongly [42]; therefore, their detachment in the migration took a longer time than a softer substrate (PDMS substrate). For human melanoma cells, similar observations regarding cell migration on glass and PDMS were reported by Chen et al. [43].

### 3.4. Effect of Chemical Properties of Substrates on Microglial BV2 Cells Migration

**Figure 7:**
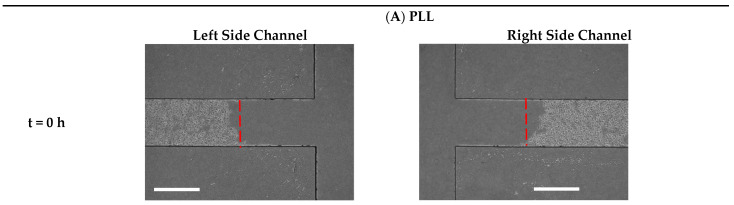

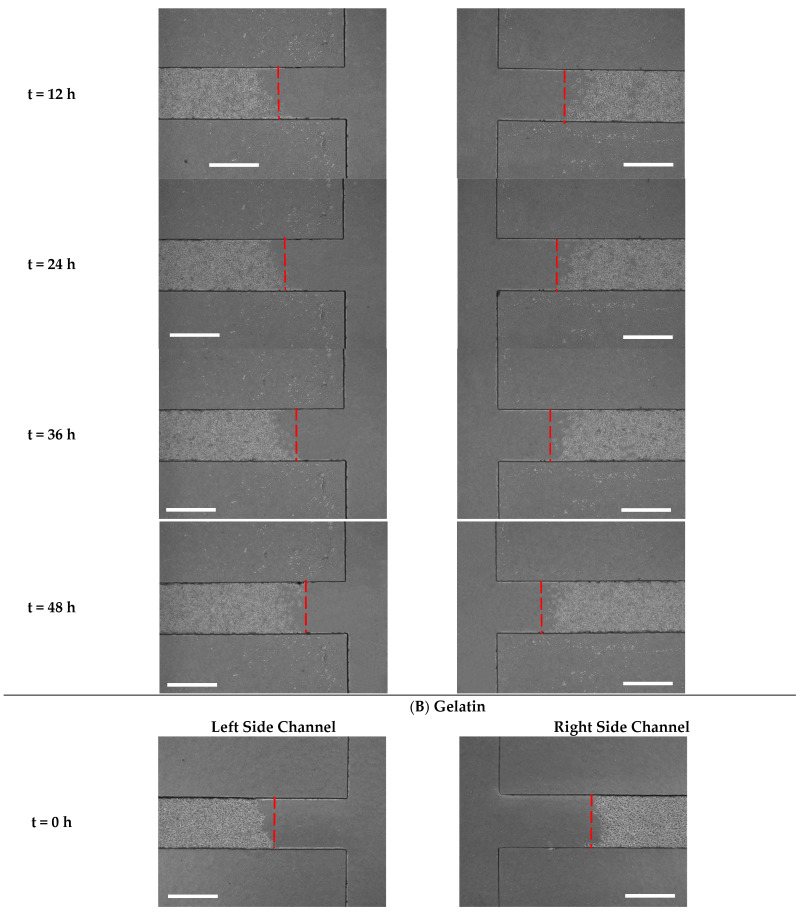

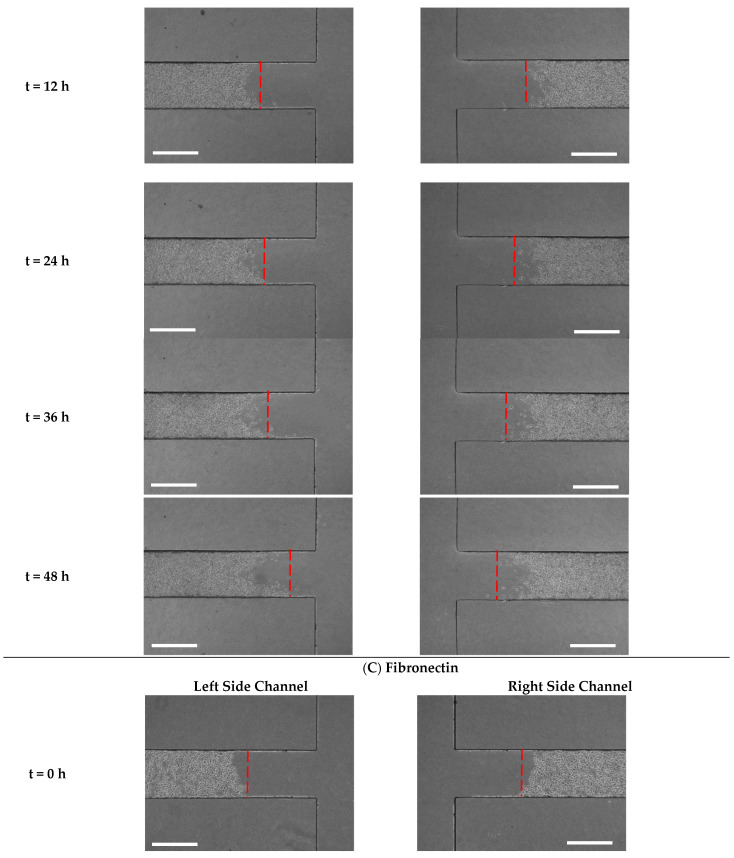

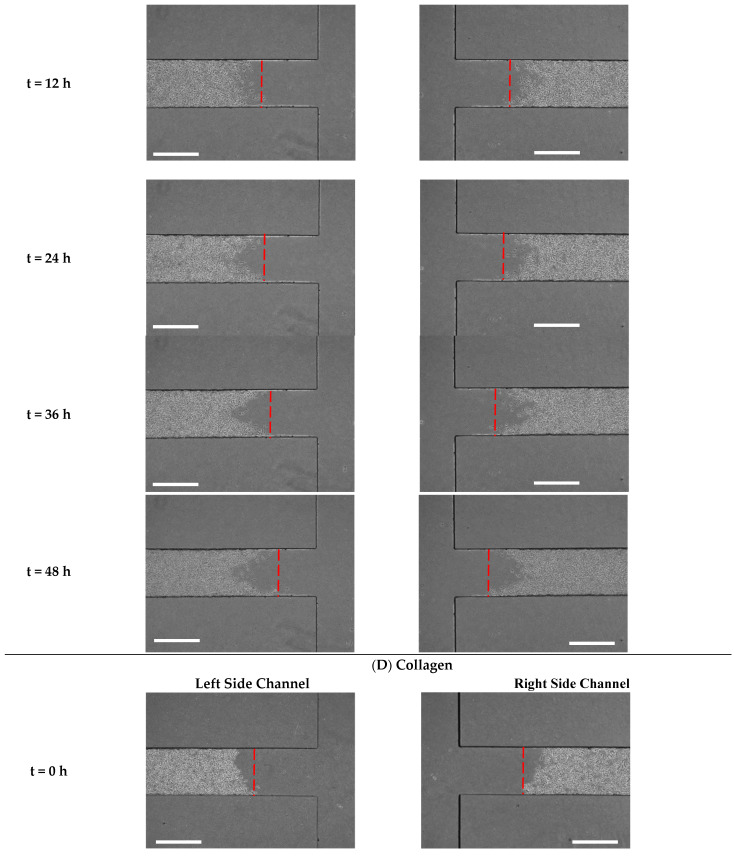

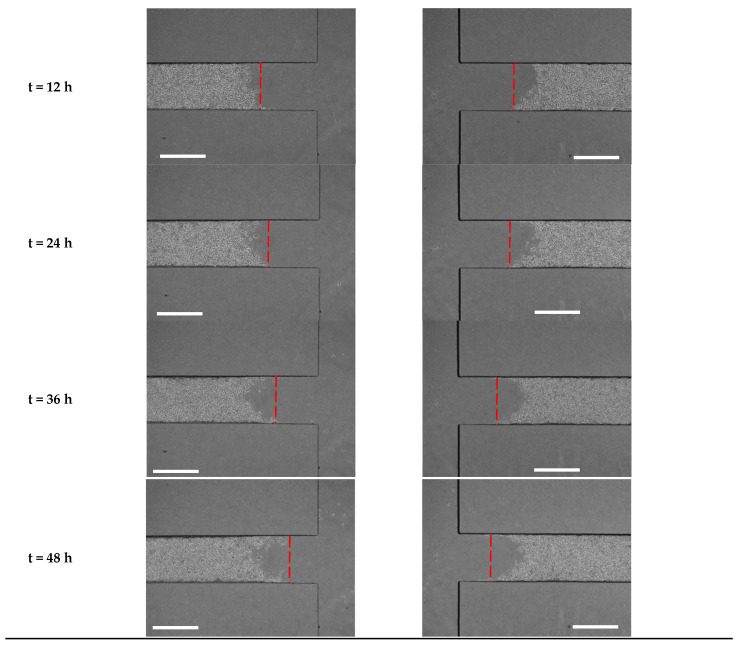
Time-lapse images from cell migration on the substrates of (**A**) PLL, (**B**) gelatin, (**C**) fibronectin, (**D**) collagen after 0, 12, 24, 36 and 48 h, scale bar = 600 µm.

To study the microglial BV2 cell mobility in interaction with ECMs, the cell migration process was quantified at 0, 12, 24, 38 and 48 h on PLL, collagen, fibronectin and gelatin (Figure 7A–D). As shown in Figure 6D, after 48 h of wound generation, the PLL and gelatin induced higher cell migration (324,107.62 ± 91,234.51 µm^2^ and 274,651.12 ± 35,718.18 µm^2^, respectively) than the control (246,651.1 ± 39,398.4 µm^2^). However, the fibronectin and collagen, with migration areas 211,994 ± 36,052 µm^2^ and 187,419.2 ± 37,860.2 µm^2^, respectively, inhibited microglial cell mobility compared to the control condition over 48 h. The investigation on the migration distance during 48 h (Figure 6E) indicated that PLL and gelatin had the highest stimulation effect (540.17 ± 152.23 µm and 457.75 ± 59.53 µm) when compared to fibronectin (353.32 ± 60.48 µm), and collagen (312.36 ± 63.10 µm). As indicated in Figure 6F, after 48 h of wound generation, the average speed of the microglial BV2 cells on PLL, gelatin, fibronectin and collagen were 11.25 ± 3.16 µm/h, 9.53 ±1.24 µm/h, 7.36 ± 1.25 µm/h and 6.53 ± 1.35 µm/h, respectively. As a result, PLL and gelatin improved the mobility of the microglia BV2 cells.

ECMs enhance the cell attachment on the tissue culture substrate by charging the substrate positively to adsorb the anionic sites on the cells [44,45]. The previous studies showed that the surface coating with ECMs either facilitated or prevented cell migration, depending on the cell type and the strategies used in the experiment [37,38]. The present study found that the migration of microglial BV2 cells was stimulated to a greater extent on a PLL-coated surface than on gelatin, collagen and fibronectin. Similarly, Mckeehan et al. [46] demonstrated that PLL increased the growth of human and chicken-like cells fibroblasts, more so than gelatin and collagen. In addition, it was observed that PLL had a positive effect on the migration of a mouse embryonic fibroblast cell line [32]. However, it was reported that using the scratch assay, collagen promoted the migration of mesenchymal stem cells more than PLL and fibronectin [47]. Klein et al. [28] presented that in the scratching method, Schwann cells migrated to a greater extent on fibronectin than PLL and collagen. For smooth muscle and endothelial cells, it was shown that gelatin-coated on a polystyrene surface had a stimulatory-migration effect [48], which supported our results, in which we observed microglial BV2 cells with the same coating on the glass substrate. The Microglia BV2 cells’ mobility was enhanced on the fibronectin-coated polystyrene substrate [49], which can positively affect cell migration [46,47]. In contrast, this result was in opposition to our finding for BV2 cell migration on the fibronectin-coated glass substrate.

## 4. Conclusions

In this present report, we developed a microfluidic wound-healing migration assay to study the effect of substrates with different chemical and physical properties on the migration of microglia BV2 cells. The microfluidic device was utilized to generate the cell-free area (wound) by using gravity as a driving force to flow the trypsin. It was shown that using the microfluidic device, despite the scratch assay, the substrate coated with fibronectin was not removed after creating the cell-free area, which enables us to quantify the cell migration in an environment closer to in vivo conditions. The study on the substrates coated with four ECMs, including PLL, gelatin, fibronectin and collagen, demonstrated that PLL and gelatin had a stimulatory-migration effect compared to the control condition (glass). Our findings demonstrated that among polystyrene, PDMS and glass substrates with different physical properties, microglia BV2 cell migration improved on the petri dish coated with plasma compared to PDMS and glass. There was a migration enhancement on the substrate with lower elasticity when their migration on the PDMS was higher than glass. The microfluidic migration assay helps us to better understand the microglia migratory behaviors in interacting with the substrates as an extracellular variable.

## Data Availability

Not applicable.
